# A Clinical and Practical Treatise on the Diseases of Children

**Published:** 1844-04

**Authors:** 


### BRITISH AND FOREIGN MEDICAL REVIEW,

FOR APRIL, 1844.

### PART FIRST.

### Analytical and Critical Reviews.

### Art. I.

*Traité Clinique et Pratique des Maladies des Enfans.* Par MM. Rilliet et Barthez.—*Paris*, 1843. Three Vols. 8vo, pp. 2408.

*A Clinical and Practical Treatise on the Diseases of Children.* By MM. Rilliet and Barthez.—*Paris*, 1843.

This is by far the most valuable work on the diseases of children with which we are acquainted. It is not, as some French treatises on this subject have been, a mere mass of rude and ill-arranged materials, collected hurriedly during a brief residence in the hospitals of Paris, but it is the result of the careful observation of two gentlemen whose opportunities for investigating the diseases of children were very considerable, and who appear to have been singularly well qualified for turning those opportunities to the best account. It was in the year 1837 that they commenced their labours, and in 1838 they published an essay on 'Pneumonia in Children,' which we noticed with its due meed of praise in our Number for October, 1841. The authors were both *internes* at the Hôpital des Enfans Malades, and as the result of a successful *concours* one of them was enabled to remain there for two years beyond the usual period of an *internât.* Their field of observation was therefore unusually extensive, while some years subsequently devoted to private practice have enabled them to correct any erroneous opinions into which the peculiarities of that field might have led them. Since the appearance of their treatise on pneumonia they have published occasional essays on other diseases of childhood, the merit of which led us to form very high expectations of what this treatise would be; but high though our expectations were, they have not been disappointed. Two circumstances confer a peculiar value upon this work: one, the fact that the authors have rejected their earlier observations, and found their statements exclusively on those made after they had acquired a certain familiarity with the subject; the other, that they in every instance state the number of cases of each disease of which they have kept a record, so that we are never left at a loss as to the degree of weight which should be attached to their statements. There is, moreover, an air of truth and good faith pervading all their observations; and they must be admitted to be much more conversant with the writings of others than most of their countrymen.

As critics, then, we shall have but little to do, and shall prefer the much more welcome task of introducing our readers to some of the many valuable observations which abound in these volumes. Still, as we trust to see the work pass through many editions, we will offer to its authors one or two suggestions calculated, in our opinion, to increase its utility. First, then, we think the nosological arrangement they have adopted is attended with many practical inconveniences, which are not atoned for by corresponding benefits. The authors have classed the different diseases under the six heads of Inflammations, Dropsies, Hemorrhages, Gangrenes, Neuroses, and Fevers; and have formed two other classes, of which the one includes Tubercular Degeneration, the other the Entozoa. The result of this attempt at scientific arrangement, founded on principles the correctness of which many will be disposed to doubt, is that very dissimilar diseases are placed in juxta-position, while those which, from seat or symptoms, often present the closest resemblance are treated of in different volumes. The table at the end of the third volume—in which the diseases are classed according to regions—does not remove, though it diminishes, this inconvenience; and in the next edition we should decidedly prefer a classification founded on regions. The appendix, too, will doubtless be removed to its proper place, as an introduction to the whole work. Our grand quarrel with the authors, however, is on the score of the extreme prolixity of their style, which has swelled out a manual for students into three closely-printed octavos of 800 pages each. We are quite convinced that by the mere curtailment of divisions and subdivisions—multiplied till, as the German proverb has it, "one cannot see the wood for the trees,"—by avoiding needless repetitions, by abridging their articles on the eruptive fevers, and by omitting the historical details—all that is really valuable might be compressed into two volumes. The articles on smallpox, measles, and scarlatina occupy 350 pages; but on subjects concerning which so much has been already written it would have been sufficient if the authors had added such remarks only as were new, or, at least, such as their personal experience had taught them to be of peculiar importance. The historical details, too, which for the most part are taken at second-hand, and often from that very incorrect author, Ozanam, add, in our opinion, to the size rather than to the value of the book. But we will now pass to a more leisurely examination of its contents.

CHICKEN-BREAST. In the introductory remarks at the commencement of the first volume, and in the appendix at the end of the third, are many valuable observations on the physiological peculiarities of childhood, both in health and disease, and on the best mode of conducting the examination of any case of illness in children. The remarks on the mode by which that deformity of the chest in childhood commonly called the chicken or pigeon-breast is produced strike us as new. Slight degrees of it are by no means unusual, and often occasion parents a needless amount of anxiety from their supposing it to be a sign of phthisis. It is, as MM. Rilliet and Barthez remark, in rickety children that the pigeon-breast is found in its most marked degree. The sternum is then extremely prominent, and of an arched form; the cartilages of the ribs run backwards, as if about to touch the vertebral column, and the axillary region is thus rendered remarkably flat. The union of the bones with the cartilages of the ribs is marked by a row of rounded prominences on either side of the chest, which are most evident about its base, and either occupy the most depressed part of the thorax or are situated a little anterior to it. This contraction of the chest exists in almost its entire length; namely, from the second or third rib to below the nipple: at this level the false ribs project and envelop the upper part of the abdomen, thus increasing the apparent depression of the chest. The size of the abdominal viscera and the intestines distended with flatus impart a spherical form to the abdomen, which is rendered still more marked by a curvature of the spine in the lumbar region, the concavity of which is directed forwards, and which diminishes the abdomen in a vertical direction.

If we watch the respiratory movements of a chest thus deformed, we shall observe a series of very remarkable phenomena, which will help us to understand the mechanism by which the deformity is produced.

.......... "During each inspiration the abdomen swells out considerably, while the chest, on the contrary, becomes contracted in such a manner as to exaggerate the peculiarity of form mentioned above; the contraction is especially well marked, a little below the nipple, as though a girdle were surrounding the chest in that situation, and forcing the abdominal organs downwards. Above this circular depression is another vertical one, reaching from a level with the axilliary region to that point where the false ribs project: and through the whole of this space, the ribs as well as the intercostal spaces become collapsed during inspiration; while the sternum is carried a little forwards and upwards. During expiration, on the contrary, the opposite phenomena occur, and the chest reverts to that state described above." (pp. 646-7.)

............ "This circular constriction which takes place at the base of the chest, and by which all its lateral parts are approximated to each other, shows plainly that the diaphragm, by its powerful and constantly-recurring action, contributes in some degree in producing the deformity. It is true that the question may be asked why the muscles which are the antagonists of the diaphragm do not prevent the collapse of the ribs, since it is necessary for the diaphragm to act effectively that the ribs should be fixed by the inspiratory muscles. It must, however, be observed, that these muscles are not inserted into the ribs at the same points as the diaphragm. Thus, the insertion of the pectoralis minor, and of the serratus magnus is behind or rather above that of the diaphragm, so that a considerable space intervenes between the attachment of these antagonist muscles. Now, so long as the costal lever continues firm, it is able to resist the traction in opposite directions to which it is subjected; but if it becomes softened in this interspace, the pectoralis minor and serratus magnus dragging it outwards, while the diaphragm draws it inwards, a constriction takes place at the weakest point, which point at the base of the chest is a little above the attachments of the diaphragm. With reference to the pectoralis major, its action is altogether different in these cases; for it is attached to the sternum itself, which it tends, in deep inspirations, to draw forwards and upwards, so that it does not in any way contribute to hinder the formation of that depression which takes place posteriorly to it. From all these muscular actions it results:

"1st. That a circular constriction of the base of the chest is formed by the diaphragm.

"2d. That the sternum is carried forwards, or more strictly, perhaps, maintained in its position by the action of the pectoralis major.

"3d. That below this circular constriction, the false ribs are forced upwards by the resistance of the abdominal viscera." (Vol. iii, p. 650.)

The vertical depression of the chest which likewise exists in these cases, is the result of the parietes of the thorax not being sufficiently strong to resist the contractility of the lungs.

This alteration in the form of the chest is a point of very considerable practical importance, since it modifies the signs furnished by auscultation, and might lead those who were not aware of it into erroneous conclusions. For very valuable remarks, on this subject, however, we must refer to vol. iii, p. 654.

An emphysematous condition of the lungs, produced by the pressure of the ribs, is one extremely frequent result of rhachitic deformity of the chest. MM. Rilliet and Barthez suggest that, in the case of adults reported to have suffered from emphysema from their earliest infancy, it is probable that this deformity of the chest had existed, and though they might have outgrown it in after-life, yet its effects on the respiratory organs were persistent. (Vol. i, p. 135.) Other causes, however, tend to produce emphysema, as bronchitis or pneumonia, or, in short, almost any affections that are attended with considerable acceleration of breathing. They deny, however, the frequency of emphysema as a result of hooping-cough, unless when complicated with bronchitis or pneumonia, since in the simple forms of the disease the conditions requisite for the production of emphysema do not exist.

"Each paroxysm consists of a series of expirations followed by one long and sibilant inspiration. Thus, on the one hand, the series of expiratory efforts almost empty the lung of the air it contained, and consequently operate in a manner directly opposed to that which is the mechanical cause of emphysema; while on the other hand the long and sibilant inspiration which succeeds the expiration, takes place under the influence of a constriction of the larynx, trachea, and bronchi, which does not allow the air to pass beyond the larger bronchial tubes. The expulsion of the air and its subsequent imperfect entrance into the air-cells during the paroxysm, are the two phenomena which account for the absence of emphysema." (Vol. ii, p. 217.)

HOOPING-COUGH. The article on this disease, though not one of the most valuable in the work, contains some extremely important observations on the diagnosis of the affection. Those who are most conversant with the diseases of children must often have been struck by the erroneous statements that parents make with reference to their children having had hooping-cough. Medical practitioners, too, are sometimes misled on this point, and pronounce that a child has got pertussis if they once hear it hoop while coughing. Not every child, however, who has been heard to hoop has, of necessity, got hooping-cough; an occasional hoop is not very unusual in ordinary catarrh, especially in children who are teething; and MM. Rilliet and Barthez point out (vol. ii, p. 224 et seq.) two diseases of very different characters, each of which may be, and often is, confounded with hooping-cough. Acute bronchitis attended with cough recurring in paroxysms is one of these diseases; the other is tubercular degeneration of the bronchial glands. The former of these affections may be distinguished from hooping-cough by the general absence of a catarrhal stage introducing the paroxysms of cough, by those paroxysms being usually shorter, less intense, often unattended with hoop, or at any rate accompanied with a very rare and indistinct hoop; and without expectoration or vomiting. It is associated from the very commencement with intense fever and accelerated respiration, a small pulse, anxious countenance, and extreme dyspnea, and tends rapidly to a fatal termination. Tubercle of the bronchial glands may be distinguished by the paroxysms of cough being usually very short, and unattended either with hoop or with mucous expectoration, or with vomiting. In its course, too, attacks of asthma often occur which alternate with the paroxysms of cough; it is frequently attended with alterations in the character of the voice, and is associated with hectic fever and night sweats, and may further be recognized by the physical signs of tubercular disease.

Stomatitis and Cancrum Oris. The subjects of stomatitis and cancrum oris are very carefully examined at vol. i, p. 260, and vol. ii, p. 149. These diseases, though essentially distinct, are very frequently confounded; an error which in our own country has been extremely common. The former is a frequent, the latter an infrequent affection; the former sometimes prevails epidemically and appears to have a contagious character; at any rate several children in the same family often suffer from it at once, while the latter occurs only in isolated cases, and is clearly destitute of all contagious property; in short, the former runs a chronic course, and has no tendency to become gangrenous, while the latter is gangrenous almost from the beginning, and runs a rapid couse to a fatal termination. The differences between the two diseases are well and concisely stated by the authors as follows:

| " *Stomatitis* | " *Gangrene* |
|---|---|
| " Begins by an ulceration, or by a deposit of plastic false membrane. | "Begins by an ulceration which is sometimes gangrenous from the first, or by an œdema of the cheek. |
| "Odour very fetid, and sometimes gangrenous. | " Odour always gangrenous. |
| "The local lesion does not extend far; the parts always preserve the same appearance. | " Extends considerably, and with rapidity; the tissues assume a peculiar blackish gray colour. |
| " But little swelling of the cheek or lips, or simply œdema of the part, without any hard portion in the centre, without tension, and without the peculiar oily appearance. | " Very extended swelling and œdema of the cheek, with a nucleus of engorged tissue in the centre; considerable tension of the parts, with an oily appearance mottled with portions of a violet hue. |
| " Secretion of saliva seldom so profuse as to run out of the mouth: when it is there is sometimes an admixture of blood, but never of the *debris* of gangrenous structures. | " Abundant salivation, constant discharge of fluid from the mouth, at first bloody, then putrilaginous. |
| " No eschar ever forms externally. | " An eschar often forms on the cheek or lips. |
| " The soft parts are never completely perforated; the bones are never denuded, the teeth seldom drop out. | " The soft parts are often perforated; the bones are always denuded; the teeth are invariably loosened, and often drop out. |
| " The course of the disease is slow if left to itself; it is cured rapidly by appropriate treatment. | " The course of the disease is rapid and invariably fatal if left to itself, often even in spite of all treatment." |

(Vol. ii, p. 149.)

In their extremely accurate description of stomatitis, they chiefly follow M. Taupin, who contributed a very valuable paper on the subject to the 'Journal des Connaissances Medico-Chirurgicales.' They recommend the application of the dry chloride of lime, or of powdered alum to the ulcerated gums, washing it off after it has remained in contact with them for a few minutes; a suggestion on which we have acted with very good result. They have met with twenty-seven cases of cancrum oris, and have thrown much new light on the morbid anatomy of the disease, of which they treat at considerable length. On examining parts which have been the seat of this affection, the whole of the cheek or lip is found swollen, shining, of a greenish or violet colour, hard to the touch, and presenting a deep-seated circumscribed engorgement. At the most prominent part of this swelling there is often a round or oval eschar, with defined edges, and varying in size from that of a pea, to that of a crown-piece. This eschar is for the most part quite dry, and of the consistence of parchment, and extends through the whole depth of the skin, but seldom involves the subjacent tissues. The mortification of the skin is not invariably met with, but the mucous membrane of the mouth is always gangrenous in a greater or less extent. Sometimes there is an elongated ulceration, with defined edges, and a blackish gray fundus, occupying the base of the lower gingivo-buccal fold, or, more frequently, that part of the internal surface of the cheek which corresponds to the interval between the dental arches. At other times the gangrenous surface is much more considerable, involving the whole or nearly the whole interior of one cheek, which is lined by a black or brown putrilage, that may be removed by scraping with the scalpel, leaving behind shreds of the mucous membrane, in which no trace of organization is discernible. The gums usually share in the destructive process, and the jawbones are in a corresponding extent blackened, sometimes necrosed, and even presenting small portions detached; while the teeth, if they have not already dropped out, may be removed by the slightest touch.

In the slightest degree of this affection, the cellular and muscular tissues of the cheek are infiltrated with serosity, but still preserve their organization unchanged. When the disease, however, has reached a more advanced stage, all the tissues are involved; those parts nearest to the mucous membrane being always the first affected. The brown putrilage mentioned above, is now found to extend four or five millimetres in depth; below it are the cellular and adipose tissue, and the muscles of the cheek all infiltrated with sanious fluid, and tending to become homogeneous, and to lose all the characters of organization, while the adipose tissue near the skin still preserves its natural texture, and is merely infiltrated with fluid. It is indeed comparatively rare to find the mortification involving the whole thickness of the cheek or lip, for usually a portion of infiltrated and hardened tissue separates the eschar of the skin from the gangrenous mucous lining of the mouth. Still, if the disease advance, a time does come when the gangrene affects the whole thickness of the parts; the eschar then becomes detached, and perforation of the cheek results.

The statements of former writers with reference to the condition of the vessels and nerves in this affection are very vague; some saying that they present nothing remarkable, while others assert that they become so confounded with the other tissues, as to render it impossible to distinguish them. To the elucidation of this subject MM. Rilliet and Barthez have devoted considerable attention; and present the following statements as the result of a careful dissection of six cases, in which this point was particularly examined:

"We have ascertained that if the tissue through which the vessels pass is simply infiltrated, but not in a state of gangrene, they are then perfectly healthy, permeable; with, perhaps, very slight thickening of their parietes; if they be running at the edge of the gangrenous parts, (we met with this condition only in one patient, and in the facial vein, having forgotten in this case to examine the artery,) they are still permeable, but their walls are thickened, and are beginning to assume the appearance of gangrenous parts. Even when they traverse the gangrenous tissues it is still possible to find them, and to trace the facial vein and artery through the disorganized parts, into the healthy structures beyond. The cavity of the vessel is then found to be occupied by a clot which reaches through the whole extent of the gangrenous parts; or sometimes the clot closes the vessel exactly at the point of its entrance into the mortified tissue, and of its exit from it, but does not extend any further in that direction; forming a cone, the base of which is situated at the edge of the gangrenous parts, while its apex reaches for some distance into the vessel where it is surrounded by healthy tissues. In either case a portion of the vessel somewhat exceeding the extent of the gangrene is removed from the circulation; its thickened walls tend to assume the colour and softness of putrefied parts; and its interior is found to contain a gangrenous putrilage. In the three instances in which we dissected the vessels in the centre of a gangrenous part, we found the arteries thus obliterated, while in two of these cases the vein was still permeable, though full of a liquid putrilage; once its parietes were extremely thickened and soft. We once examined the condition of the nerves, and found that in the midst of the gangrened parts they assumed the colour and appearance of the other tissues. This change, however, had not reached beyond their exterior; their neurilema was gangrenous, but the pulp of the nerves had preserved its natural colour and appearance, and seemed to have resisted the mortification. Once we traced the whole course of the duct of Steno; it continued permeable in the midst of the gangrenous tissues, assuming their appearance where it crossed them, and opening into the mouth by a free orifice in the midst of the putrefied remains of the mucous membrane." (Tome ii, p. 132-3.)

Their investigations lead them to the opinion that cancrum oris always commences in the mucous membrane of the mouth; though they would not deny the possibility of its commencing in the tissues intermediate between the skin and mucous membrane, since the high authority of Richter asserts that to be sometimes the case. The condition of the vessels they regard as a consequence, not as the cause, of the gangrene. For their faithful portraiture of the symptoms of the disease, we must refer our readers to the work itself; since many other interesting topics demand a notice.

Croup. The chapter on croup is enriched by an essay on tracheotomy from the pen of M. Trousseau; which is extremely valuable as embodying the results of the experience of a man who has performed the operation 121 times. It is customary in our own country to regard tracheotomy as an operation in itself extremely hazardous, and consequently to defer its performance in cases of croup until all other remedies have been had recourse to without avail. M. Trousseau combats this opinion, and appealing to the favorable results of the operation when performed for the removal of a foreign body from the trachea, as contrasted with its fatality when practised for the relief of chronic inflammation of the larynx, he concludes that it is not in itself dangerous; though it may become so when its performance is delayed till after symptoms of asphyxia have existed for a considerable time. He infers therefore that in all cases of croup, the operation should be performed so soon as we can feel tolerably certain of the presence of false membranes in the larynx. By so doing two advantages are secured, for we do not have to contend against the altered state of the blood, and the engorgement of the lungs and cerebral congestion which exist in advanced stages of croup; and secondly, the false membranes will probably be found to be less extensive, and the applications resorted to for their removal will be more efficacious. He next discusses the comparative merits of tracheotomy and laryngotomy, and decides in favour of the former; partly because by it the air-passages are opened lower down, and the chances of arresting the extension of the false membrane to the bronchi are thus increased. Another reason in its favour, and to which he attaches greater weight is this, that the canula which it is indispensably necessary to introduce into the air-passages, usually excites violent inflammation of the edges of the wound, and almost always induces the necrosis of the cartilages in its immediate neighbourhood. This occurrence is of small importance when it involves the rings of the trachea, for the necrosed portions can be easily removed, and their loss does not cause any contraction of the tube; but far more serious consequences would result from the larynx being thus affected, since after the cure of the croup, the patient would have to undergo all the various evils produced by necrosis of the thyroid and cricoid cartilages. There would be their chronic inflammation and suppuration to combat, with much reason to apprehend that the larynx might remain permanently deformed, or that the tumefaction of the mucous membrane might cause dyspnea as serious as that which the croup had produced, or at least that the voice would ever after be seriously impaired. With reference to the greater danger that is supposed to attend tracheotomy, M. Trousseau remarks that of 121 patients on whom he performed this operation, only one died during its performance, and in that instance the patient died in a fit before the first incision was completed.

It is M. Trousseau's custom in performing the operation, if he finds it impossible to avoid the thyroid veins by careful dissection, to divide them without tying, since the hemorrhage always ceases on the introduction of the canula; while tying them not only lengthens the operation, but is attended with some danger of inducing phlebitis. As soon as he has completed the incision into the trachea, he introduces an instrument to keep the edges of the wound apart, raises the child from the recumbent posture, and waits for a few moments till the hemorrhage ceases. He then, if the case is very urgent, immediately introduces the canula; if the symptoms are less pressing, he allows the dilator to remain for some time, in order to afford opportunity for the expulsion of the false membranes; while he further endeavours to favour their discharge by dropping water into the bronchi, and by sponging out the trachea repeatedly. He prefers the curved canula of M. Bretonneau, and the bivalved canula of M. Gendron to any others hitherto invented, though for adults and for all children except those who are very young, it is advisable to use a double canula. This ought to be sufficiently long to enter to a depth of two centimetres into the trachea; for by the second day after the operation; the swelling of the soft parts around the wound will prevent it from entering more than five or six millimetres, and then if the canula were not sufficiently long, it might be displaced during the efforts of coughing. As often as the respiration becomes difficult, the canula must be removed, if there be any reason for referring the dyspnea to any obstruction in the tube. Usually it is sufficient to change the canula twice in twenty-four hours; when a double canula is employed, the inner one must be removed every three hours, which may be done without causing the slightest annoyance to the patient. At the time of withdrawing the canula, the dilator should be introduced, and while the edges of the wound are thus kept apart, the trachea should be sponged out, and the medicated applications employed. In the course of two or three days the use of the dilator becomes unnecessary, the edges of the wound continuing open of their own accord. It is a matter of great importance to withdraw the canula at as early a period as possible. If the case should advance favorably, the same sedulous care to keep the canula free becomes unnecessary after the fourth or fifth day; and if the air should appear to pass at all by the larynx, a canula may be introduced incompletely closed by a little plug of cork. If respiration still go on well, the caliber of the canula may be reduced every day until it is finally removed, which may usually be done from the tenth to the thirteenth day. Once he succeeded in removing it as early as the fourth day, and once he was compelled to allow it to remain until the fifty-third day.

M. Trousseau does not rely for success solely on the opening the trachea, and introducing the canula, but he attaches great importance to the employment of topical remedies. These consist in dropping into the air-passages, fifteen to twenty drops of a solution of about five grains of nitrate of silver in an ounce of distilled water, and at the same time cleansing out the trachea with a sponge dipped in a solution of one part of nitrate of silver in five of water. This process he repeats four times the first day, three times the second, once or twice on the fourth, and then suspends its employment altogether.

He concludes with some interesting remarks on the prognosis in these cases which we would fain extract, but must content ourselves with referring to them at page 379 of vol. i. The results of this plan of treatment naturally excite our curiosity. They are by no means brilliant. M. Trousseau has operated on 112 croup cases, in 27 of which the patients survived; and if to these we add those cases in which other surgeons have pursued a similar plan, we obtain a total of 150 cases; 39 of which, or rather more than one quarter, had a favorable termination. It cannot, however, with any fairness be assumed that in all of these 39 cases, the patient's life would have been sacrificed but for the operation. The very reverse of this, indeed, is probable, for the old pupils of M. Bretonneau, of whom M. Trousseau is one, often resort to the use of tracheotomy in cases of croup, without employing any previous treatment with the exception of the local application of caustics.*

Cerebral Hemorrhage. The second volume contains a very valuable essay on cerebral hemorrhage in children, a subject that has not re-

* Some sensible remarks on this premature employment of tracheotomy are contained in the Bullétin Général de Thérapeutique for October, 1842, on occasion of two cases operated on without any previous treatment, related in the Journal de la Société Médicale d'Inde et Loire.

ceived all the attention which it deserves, since many peculiarities distinguish the different forms of this disease in the child, from the same forms in the adult. Hemorrhage may occur in all situations within the cranium; as between the skull and dura mater, between the dura mater and arachnoid, into the cavity of the arachnoid, into the tissue of the pia mater; or into the substance of the brain, or the cavity of the ventricles. Of all these forms the most frequent, and from its frequency the most important is the hemorrhage into the cavity of the arachnoid. It has nevertheless been overlooked by many writers, and but slightly noticed by others; probably in some instances owing to the fact that the effused blood undergoes changes which assimilate it in appearance to false membrane, for which it has been mistaken by some observers. MM. Rilliet and Barthez base their remarks on twenty observations, seventeen of which came under their own notice, while for the particulars of the remaining three, they are indebted to a friend. From an examination of these cases it appears, that pure, unchanged blood, is seldom found in the cavity of the arachnoid; it usually undergoes rapid alterations; the serum separating from the crassamentum, while the latter becomes by degrees converted into a thin, elastic, false membrane, which sometimes resembles the arachnoid; at other times closely resembles a fibrous membrane. The first form in which the clot is found is that of a dark-red, almost black, coagulum, of varying extent, usually thicker at the centre than the circumference, adherent to the arachnoid, (almost always to its parietal layer,) but easily detached from it, and leaving the serous membrane, with which it had been in contact, smooth, polished, and unaltered. Sometimes there is but one clot; at other times there are several; in either case the edges often extend into a thin, yellow, or transparent false membrane; so thin indeed that at first sight its edges cannot be distinguished, and it might be confounded with the arachnoid, and lead to the supposition that the effusion of blood had taken place between the arachnoid and dura mater, if it were not found that the clot and false membrane are removed together. This continuity of substance between the clot and false membrane points out the common origin of the two, and warrants the conclusion that the latter is identical with the former; its apparent difference being merely the result of the absorption of the colouring matter of the blood. This is most clearly seen to be the case, whenever portions of coagulum are interspersed through different parts of the false membrane. Clots of a deep-red colour, a thick very lacerable membrane of a reddish-yellow colour, and infiltrated with serum, and a thin, more transparent, and less coloured false membrane, then, make up one continuous layer. It often happens that in the course of time, the thin, delicate, false membrane grows opaque and resisting, and, assuming a pearly lustre, altogether loses its resemblance to the arachnoid, but presents instead considerable similarity to the dura mater. This change is probably brought about by the deposition and subsequent alteration of successive layers of blood; at least MM. Rilliet and Barthez have found membranes of this kind presenting a distinctly-stratified structure in the adult, though they have not met with any well-marked specimen of it in the child. Clots and these false membranes for the most part coexist, and are found usually on the convex surface of the brain, sometimes on its plane surface also, but never on that alone. They are generally present on both hemispheres, and do not occur on one side more frequently than on the other. Sometimes they are perfectly dry, but in the majority of cases the cavity of the arachnoid contains some fluid, which varies much in colour. This fluid is seldom present in any large quantity, and the cases in which it is abundant are those of very young children, in whom the ossification of the skull is incomplete. In one instance of this kind, the arachnoid cavity contained nearly a pint, in another, nearly a quart, of fluid, and such an occurrence constitutes one form of chronic hydrocephalus.

The symptoms of the affection are extremely obscure, except when the effusion of fluid gives rise to hydrocephalus, when diagnosis is aided by the sensible enlargement of the head. In this case, however, though important, it is often very difficult to distinguish between chronic hydrocephalus arising from other causes and that which proceeds from sanguineous effusion, since the hemorrhage may occur at different times, and the enlargement of the head may consequently take place gradually. The symptoms of ordinary chronic hydrocephalus develop themselves more slowly than those which result from meningeal apoplexy, and it will likewise help diagnosis if further observation should substantiate the authors' statement, that meningeal apoplexy never occurs in children more than two years old, while chronic hydrocephalus from other causes is by no means rare above that age.

Hemorrhage into the substance of the brain, so frequent an occurrence in the old subject, loses much of its importance in the child. It does not happen half as often as hemorrhage into the cavity of the arachnoid, and is of comparatively small moment, being generally a secondary phenomenon, supervening in the course of some disease which would in itself prove fatal to life, and taking place only a few days before death. Sometimes, too, it is completely latent, and the morbid anatomist is the first to discover the existence of a lesion, which had escaped the observation of the practitioner. It may occur as capillary apoplexy, or a circumscribed extravasation of blood may take place ; but it is seldom that either form exists uncomplicated with tubercular deposit, or meningitis, or some other disease of the brain. Its symptoms when it occurs in the idiopathic form are most various and uncertain, and altogether unlike those which characterize apoplexy in the adult; while apoplectic symptoms have existed in some instances during life, where a post-mortem examination has failed to discover any trace of effusion of blood. In the secondary form, too, the symptoms are not more decisive.

Perhaps we cannot go further in the differential diagnosis of the various forms of cerebral hemorrhage, than the mere statement that convulsive symptoms more frequently attend hemorrhage into the membranes, and inflammatory symptoms hemorrhage into the substance of the brain. The diagnosis of cerebral hemorrhage from other affections of the brain is scarcely more easy; for the convulsive form may be confounded with idiopathic convulsions or with those dependent on the presence of cerebral tubercles; the inflammatory form with certain cases of softening of the brain and encephalitis or meningitis; when attended with paralysis that symptom may be referred to softening of the brain, and the hydrocephalus which sometimes results from it may be confounded with ordinary chronic hydrocephalus of the ventricles of the brain.

Among the causes of cerebral hemorrhage there are scarcely any more influential than obstruction to the circulation, however produced. Hence compression of the superior vena cava by enlarged bronchial glands, or of any of the large venous trunks by enlargement of some of the abdominal viscera has a strong tendency to induce it. Sometimes it is the result of disease of the sinuses of the dura mater, at other times it follows the injudicious repulsion of eruptions on the scalp. Occasionally it occurs suddenly, and in children in perfect health ; but usually it is associated with a debilitated state of the system, and is very often connected with tubercle, the deposit of which in the brain suffices in many cases for its production without the concurrence of any other cause.

Tuberculous Disease. There are many more subjects treated of in the first two volumes on which we had wished to lay MM. Rilliet and Barthez's remarks before our readers, but we must forbear, for the third volume, which is wholly occupied with an account of the different forms of tuberculous disease, requires, even for the most cursory analysis, far more space than we can allot to it. The first 160 pages are devoted to general observations on tuberculous disease, which the authors assert, in opposition to the opinion of many writers of celebrity, to be identical with scrofula. In support of this opinion they allege, that having examined the bodies of a very large number of children at the Hôpital St. Louis and Hôpital des Enfans, they never met with a single instance in which tubercle was not present in some organ or other. They propose, therefore, rejecting the term scrofula from medical nomenclature altogether, and substituting that of tuberculization.

After some very interesting details with reference to the morbid anatomy of tubercle, we find the authors, at p. 46, distributing the 312 cases that form the basis of their remarks into three categories: of which the first, comprising more than the half of the cases, includes all those instances in which death was evidently attributable to the tubercular degeneration; the second, to which about one fourth of the cases may be referred, comprises those in which the extent of tuberculous deposit was inconsiderable, and evidently not the cause of the fatal event; and the third comprises those instances in which the amount of tubercular deposit was intermediate between the other two.

The authors find, on examining these three classes with reference to sex and age, that there is but little difference between them in respect of the former, but that abundant tubercular deposit is much oftener met with from six to fifteen than from one to five years of age; that the mean amount of tubercular deposit is oftenest found from one to two, and next in frequency from three to five; and that from three to five and a half years of age very scanty tubercular deposits are more frequently found than at any other period of childhood. In classing the different organs according to the frequency with which they become affected by tubercle, they arrange them in the following order: the lungs, the bronchial glands, then, at a great distance beneath them, the mesenteric or abdominal glands and the small intestines. After these organs come the pleuræ and spleen, then the peritoneum, the liver, the large intestines, the membranes of the brain, the kidneys, brain, stomach, and pericardium. Very nearly the same order would be observed in a classification of the organs according to the extent of disease in each; for those which are oftenest the seat of tubercle are usually the seat of its most abundant deposit. The same order, too, would hold good with reference to those organs in which tubercular deposit deposit most frequently coincides with the same disease in other parts. Tubercle is not unfrequently found exclusively confined to the thoracic organs, and it is not very unusual to meet with it only in the abdominal viscera. It sometimes exists in the brain, and nowhere else; but it is very rare to find it in the brain and abdomen but absent from the chest.

The presence of tubercle in the system betrays itself by very different symptoms, according to the organ which is its seat. It may, however, be stated generally that it runs either an acute or a chronic course, and that the acute form is either simple or assumes a typhoid character. The acute form is usually found, on a post-mortem examination, to be associated with the presence of gray or yellow granulations, or with small, isolated, miliary tubercles, seldom with softened or cretaceous tubercle, and seldom with other than very small cavities in the lung. Sometimes, however, large masses of tubercle are met with, which by their rose-coloured hue indicate their recent formation. The tubercular deposit in this form is usually general, though one organ in particular may be its especial seat. When the symptoms assume the typhoid character the prostration of strength is very marked, and the child lies almost in a state of stupor. The gums bleed, and they, as well as the teeth, are incrusted with sordes; the tongue is seldom universally moist; usually it has a great tendency to become dry, while at the same time its edges, or even its whole surface, are red; and the abdomen is large, tympanitic, and tender on pressure.

"Lenticular spots are seldom present; or if they exist they are but few, not very apparent, and do not persist long. The delirium may be severe and of long continuance; at other times the oppression is very great, although auscultation may not detect any or but few symptoms of thoracic disease. These typhoid phenomena may last during the whole course of the disease; but sometimes they appear during the course or at the termination of the simple acute form, when they last only a few days." (Tome iii, p. 75.)

The duration of the acute form of tubercular degeneration varies greatly; a fatal termination sometimes occurring in sixteen or eighteen days, at other times not till after sixty or eighty from the first manifestation of the symptoms. Tubercular deposit in the membranes of the brain runs its course most rapidly, and that which involves the lungs most slowly. General tubercular deposit, without predominant affection of any one organ, runs its course, in the majority of cases, in from twenty-five to fifty days.

The chronic form is characterized anatomically by the presence of all the different forms of tubercular deposit; by softened tubercles and tuberculous cavities. The local symptoms attending it are much more marked than in the acute form, and usually indicate tolerably plainly the organ which is the chief seat of the disease. It generally runs its course in from three to seven months.

Bronchial Phthisis. The authors were not placed in such circumstances as to enable them to throw much additional light on the causes of tuberculous disease. We therefore pass on to the subject of bronchial phthisis, to the symptomatology of which, especially, they have made a very valuable contribution. The essays of previous writers on tubercle of the bronchial glands are valuable almost entirely for their anatomical details; and in our own country the disease has been passed over almost without notice, except by Sir James Clark, who devotes some pages to it in his work on Consumption. But, neglected though it has been, it merits the most careful examination, since it is one of the gravest and most frequent maladies incidental to childhood.

Tubercle may be deposited in almost any form in the bronchial glands, but its deposit most frequently takes place as tubercular infiltration, and usually commences in the centre of the gland. When the whole of the gland has become involved in the disease its size is considerably increased; and glands exterior to the substance of the lung often become as large as a chesnut, but those imbedded in its tissue never exceed the size of an almond. The tuberculous matter is inclosed by a thin cyst, the inner surface of which is seen to be supplied with a delicate network of vessels; but as the tubercle softens the parietes of the cyst grow thicker, and may after a time be divided into two layers. This softening occurs at various periods: it for the most part commences at the centre of the gland, but sometimes it begins at its periphery, and in some instances the changes seem to take place simultaneously in its whole substance. The softened tubercle is generally discharged from the system by the formation of a communication between the cyst and surrounding organs, but in some very rare cases the contents of the cyst become absorbed, and it is found perfectly empty. Another change which may take place is the transformation of the tubercle into cretaceous matter; it almost invariably happens, however, that a small part only of a diseased gland is thus transformed, the tubercle in the rest of its substance remaining unaltered. While these morbid processes are going on in the glands themselves, one of two effects may be produced on the neighbouring parts: either they are compressed by the gradual enlargement of the glands, or intimate adhesions form between them and the glands, and perforation of their substance eventually takes place. The large glands may compress the vessels, or the air-tubes, or the pulmonary tissue. Serious results, however, from pressure on the vessels occur less frequently than might have been expected; and MM. Rilliet and Barthez have not met with an instance in which anything approaching to obliteration of the bronchi had been produced by the pressure to which they had been subjected. Compression of the trunk or branches of the pneumogastric nerve is a frequent occurrence; associated with which the writers have observed a spasmodic cough, resembling hooping-cough, (on the diagnosis of which we have already quoted their observations,) various modifications of the voice, and a diminution of it amounting to almost total aphonia; and twice they witnessed very distressing attacks of asthma result from it, but in no instance have they observed it produce spasm of the glottis, as in the cases recorded by Dr. H. Ley.

Instances are on record in which the œsophagus and the large vessels of the chest have been perforated by tubercular bronchial glands; but the air-tubes are, beyond comparison, most frequently subjected to this lesion:

"The bronchi being unable, like the vessels or the œsophagus, to escape compression, it often happens that adhesions more or less intimate form between them and the surrounding glands. These adhesions are formed through the medium of a cellular tissue, which, though originally loose, becomes daily denser and denser. Sometimes, too, vessels sufficiently large to be seen by the naked eye form a communication between the gland and the bronchus. At this period the gland may still be detached, and the external surface of the bronchial tube with which it was in apposition will be seen vividly injected. In course of time this adhesion becomes so intimate that it is impossible completely to remove the gland, and the surface of the bronchus beneath it is found, in a few cases, to present a marked depression." (Tome iii, p. 171.)

This union between the two organs being once established, a series of changes take place, which result in the softening of the tuberculous matter, and in the communication of the interior of the cyst with the bronchial tube; but the mode by which the communication is effected is not in all points satisfactorily ascertained. The extent of the perforation of the bronchus for the most part bears a direct proportion to the degree of softening of the tubercle; but the occasional occurrence of perforation of the tube while the tubercle in the diseased gland is in a state of crudity, would lead to the inference that it may result from interstitial absorption, produced by the long-continued pressure of the diseased gland. Various indeed as the appearances are which perforated bronchi present, it may be doubted whether the perforation is not always the result of the same process, namely, of interstitial absorption from pressure. According to MM. Rilliet and Barthez—

"The character of the perforations varies according to whether they are of recent or of old standing; whether they communicate with a cyst filled with softened tuberculous matter, or are in contact with tubercle in its crude state. In the latter case the edges of the perforation are sharp, nearly circular, and usually present no traces of injection. In the other case, on the contrary, the wall of the bronchus in contact with the cyst is of a bright red, worn from without inwards, and the perforation presents irregular edges, in which some remains of the cartilaginous rings may be distinguished." (Tome iii, p. 171.)

Glands most variously situated may thus communicate with the bronchi. Sometimes the glands remote from the lung and surrounding the large bronchial tubes communicate with their cavities; at other times the bronchi are perforated by glands situated in the substance of the lung. In this case "a communication seems to be established between the substance of this organ and the cavity of the gland, and a section dividing these two parts shows the parenchyma of the lung traversed by cavities the walls of which are formed by carnified pulmonary tissue. These cavities, which communicate freely with the bronchial cyst, are lined by a false membrane, exactly similar to the red, uneven, thick lining of the cyst itself." (Tome iii, p. 173.) The nature of these cavities has been disputed, for it is possible that they may be formed by a destruction of the pulmonary tissue itself, though the opinion of the authors seems to us more probable—that they are produced by the softening of small subdivisions of the tuberculous gland which had been imbedded in the substance of the lung.

Serious difficulties attend the investigation of the symptoms which accompany this disease. These difficulties arise in great measure from the circumstance that tuberculization of the bronchial glands is seldom met with alone, but is for the most part associated with similar changes of the lungs and pleura, which render the symptoms complex and uncertain.

We do not know that we can present the symptomatology of bronchial phthisis in a clearer or more condensed form than that in which it has been done by the writers themselves:

"The bronchial glands, when tuberculous, form a more or less voluminous tumour, that interferes with the functions of the different organs with which it is in contact. Thus, by compressing the superior vena cava, they may occasion, 1, œdema of the face; 2, dilatation of the veins of the neck; 3, a livid colour of the face; 4, hemorrhage into the cavity of the arachnoid.

"From the compression of the pulmonary veins there may result, 1, hemoptysis; 2, œdema of the lung. When the glands compress the pneumogastric nerve there may arise, 1, various alterations in the tone of the voice and of the cough; 2, fits of coughing similar to those of hooping-cough; 3, attacks of asthma, which, under other circumstances, are most unusual in children.

"The action of the glands on the lungs and bronchi is extremely remarkable. By compressing the air-tubes they occasion, 1, Loud, very persistent, sonorous râles, which sometimes are very singular in their tone and character; 2, they impede the circulation of the air, and thus occasion obscurity of the respiratory sound. This phenomenon may also depend on the œdema which is produced by the compression of the pulmonary vessels.

"The action of the glands on the bronchi, however, is not confined to the compression of those tubes, but they serve also as conductors of the vibrations of sound. Hence result the following phenomena. 1st. Although the lung may be almost or altogether healthy, various modifications of the respiratory sound may yet be heard in different parts of the chest, such as prolonged expiration, bronchial respiration, and all those sounds which in the normal condition are found in the bronchi, but not transmitted to the ear. 2d. These phenomena are still more striking if there should also exist certain pulmonary lesions, since their stethoscopic signs, even though usually not very marked, will appear to be exaggerated by the presence of the tuberculated glands. Thus crude miliary tubercles may under these circumstances give rise to bronchial or cavernous respiration, or to pectoriloquy; and if they have begun to soften, or are attended with slight bronchitis, distinct gurgling may be perceived. 3d. The stethoscopic sounds arising from the lesion of one lung may be transmitted to the opposite side, and thus excite a suspicion of the existence of a double lesion. 4th. The bronchial glands, in short, owing to their lying in contact with the vertebral column, while they also surround the bronchial tubes, transmit directly to the ear all sounds both natural and abnormal which may be found in the lung at points remote from the walls of the chest, and thus seem to exaggerate them. 5th. It is especially at the upper and posterior part of the lungs that these stethoscopic phenomena are usually detected, and it is much less common for them to be heard at the front of the chest.

"All the symptoms just enumerated and which result from the action of the enlarged and hardened glands on the vessels, nerves, bronchi, and lungs, are not constantly present, nor do they all exist at the same time, for their production depends on the situation of the glands and on their enlargement taking place in a particular direction. But, moreover, in cases where these phenomena do exist, they are all liable to very remarkable intermissions; thus, the œdema of the face alternately appears and disappears; the livid colour of the surface is not constant; the changes in the tone of the voice and cough, the paroxysms of coughing, and the attacks of asthma occur on one day and cease on the following day; to recur again after the lapse of a longer or shorter time.

"The stethoscopic signs do not continue always the same, nor do they progressively increase in degree: but bronchial respiration will be heard on one day, while on the morrow nothing more will be perceptible than prolonged expiration, and again on the day following the breathing will have resumed a cavernous character; so that feeble respiration, prolonged expiration, bronchial breathing, cavernous respiration, pectoriloquy, gurgling, and even rhonchus may alternate with or succeed to each other at various times without any regularity. These variations depend sometimes on the frequency, sometimes on the extent of the respiratory movements, or on the degree of the pulmonary lesion. In most cases, however, there are doubtless many other concurrent causes, of which we are ignorant; for the morbid phenomena that result from the compression of organs by tumours are usually intermittent.

"If the bronchial tubercles are softened and communicate with the bronchi, all the symptoms just enumerated do not exist, because the tumours which are generally smaller are situated in the substance of the lung, and are no longer in contact with the vertebral column: hence neither cavernous breathing nor gurgling are any longer audible, unless tubercular cavities exist in the lung itself.

"The character of the expectoration is seldom or never of any great service in assisting us to form a diagnosis.

"The phenomena which may be observed result almost entirely from the ulceration and perforation of the organs with which the glands are in contact, and in the present state of our knowledge we are not acquainted with anything capable of indicating that they depend on the glands and not on the lung. Thus perforation of the lung occasions pneumothorax, that of the pulmonary vessels furious hemoptysis, and the formation of a communication between the œsophagus and the bronchi or trachea by the bronchial glands, may occasion violent paroxysms of cough whenever the patient attempts to swallow liquids." (Tome iii, pp. 198-201.)

The results of percussion vary much; perhaps it yields no sign so frequently as increased dulness of the interscapular region, but this so often corresponds with various alterations in the respiratory sounds that it may give rise to a suspicion of the existence of pulmonary phthisis. Two circumstances, however, will generally suffice to preserve from error: first, the fact that in bronchial phthisis the diminution of resonance is permanent, while the results of auscultation differ at different times; and second, the want of correspondence between the indications furnished by auscultation, and the extent and degree of the dulness; so that, for instance, bronchial respiration may be heard in a situation where the resonance is but very slightly diminished.

Valuable as these remarks are, they still leave the subject in much obscurity, though no better rules for diagnosis could be laid down than are contained in the following paragraph:

"If we observe cough, emaciation, fever, and sweats occur in a child from three to four years of age, without our being able to detect any physical signs of tubercle in the lungs, or to discover any indication of tubercle either in the brain or the abdomen, we may then suspect its existence in the bronchial glands. This presumption would amount to certainty, if we were to notice an alteration in the character of the cough, such as its occurring in short paroxysms, but without being attended with hooping or vomiting, or its becoming hoarse; if we were to hear large ronchus in the trachea, or very persistent sonorous or sibilant *râle*, if we were to observe asthmatic attacks, or very marked but intermittent changes in the voice, or œdema of the face, provided that we had ascertained that this latter phenomenon did not depend on some other cause, such as disease of the kidneys. Great attention also ought to be paid to the signs furnished by auscultation or percussion. As has already been mentioned, they are remarkable for their intermittent character. It is especially the changeableness of the signs yielded by the use of the stethoscope compared with the persistence of those elicited by percussion, which must form the basis of our diagnosis. Great importance must also be attached to the situation in which changes of the respiratory sounds are detected. The signs of bronchial phthisis are perceptible almost exclusively at the upper part of the lung, and principally in the interscapular space, on a level with the root of the bronchi; they are also occasionally, but much less often, perceptible in front. In adults such a distinction would perhaps be of no great service, since in them tubercular deposit occupies almost exclusively the summit of the lung, but this is not the case in the child, but the tubercles are often scattered through various parts of the lung, or if they occupy its upper part auscultation beneath the clavicles will reveal their existence. Whenever, therefore, in a child affected with some chronic pulmonary complaint, the signs of tubercles are discovered in the interscapular space, there will be reason to conclude, if they vary in their degree and fluctuate in their progress, that they depend on tubercular degeneration of the bronchial glands." (Tome iii, p. 204.)

PULMONARY PHTHISIS. They next pass to the investigation of tubercle in the lungs of children ; and here we may notice a very important remark with reference to the stethoscopic signs of their existence, the truth of which we can fully confirm from our own experience. It will be found that some of those physical signs from which we should without hesitation conclude that tubercular degeneration of the lungs existed in the adult, do not always indicate the same serious mischief in the child. Thus, for instance, that unusually harsh respiration which in the adult is so valuable a sign of the presence of crude tubercle in the lungs, loses much of its value in the child from the circumstance that it may exist even when the lung is perfectly healthy ; and that it often is not sufficiently confined to one spot to warrant our drawing any conclusion from it. The subclavicular region is that in which this coarse respiration is most frequently heard, even in children whose lungs are perfectly sound ; in the same situation, too, the expiration is often considerably prolonged, without there being any pathological condition which could account for this phenomenon ; and great doubt is thus thrown over two of the most valuable indications of the early stage of phthisis.

The occurrence of severe pneumonia in the course of phthisis appears to be more frequent in the child than (judging from the remarks of M. Grisolle and M. Louis) it is in the adult. It may supervene as a complication either of the acute or chronic form of phthisis. In the former case it is sometimes coeval with the earliest symptoms of tubercle, but oftener it does not occur until after such symptoms have existed for some time. In the latter case intense fever and heat of skin, with a full and hard pulse and all the indications of acute inflammation suddenly come on. The cough, previously slight, becomes all at once very frequent, the oppression of the breathing is intense, and auscultation detects moist sounds through the whole posterior part of the lung, or perhaps even bronchial respiration. After these symptoms have existed for some time either the patient dies or a degree of remission takes place in the symptoms, which, however, continue in the main the same. Fever is still present, but the pulse has become small, the face has grown pale, and the child, who before the attack was not all emaciated, loses flesh rapidly. Auscultation continues to yield the same results ; moist sounds are present in abundance ; the bronchial respiration, dulness, &c. vary in degree, but do not altogether disappear, and the patient dies in two or three months. Sometimes the pneumonia is very extensive from the outset, and then it proves fatal much more rapidly ; but usually successive attacks take place, the hepatization of the lung gradually extending with each recurrence of the inflammation till it proves fatal.

When pneumonia supervenes on the chronic form of phthisis, it usually terminates in death after a few days, seldom lasting longer than a fortnight. A sudden exacerbation of all the symptoms is observed accompanied often with a tinge of blood in the expectoration, and with pain of a pleuritic character. The moist sounds which before might have been heard over a small surface now become more abundant and more extensive, bronchial respiration is perceived through the whole posterior part and base of the lung, and death ensues after some days.

There is besides a latent form which pneumonia assumes in some of these cases, that hardly betrays itself by any symptoms. The patients gradually lose their strength, and become unable to leave their bed, but without any great increase in the fever, cough, or oppression, though the prostration of their strength is very marked. Auscultation is probably neglected from the fear of distressing the patient; and when he dies, after the lapse of a few days, very extensive pneumonia is discovered, the existence of which had not even been suspected.

Mesenteric Phthisis. The error which regards the tabes mesenterica as an extremely common disease of childhood is by no means confined to the vulgar. A similar opinion is expressed by many authors, who have not only confounded a number of different affections—as tubercular peritonitis, enteritis, ulceration of the intestines, &c.—under one common name, but have also regarded as morbid the large abdomen natural to childhood. The presence of slight tubercular deposit in the mesenteric glands is, it is true, far from being a rare occurrence, since MM. Rilliet and Barthez found it in half of their post-mortem examinations of tuberculous children. The number, however, in which this tubercular deposit was so considerable as to be of much moment was far smaller, not exceeding one in sixteen. MM. Rilliet and Barthez have satisfied themselves that serious mesenteric disease hardly ever exists in children under three years of age, that the affection is always slight in proportion to the youth of the children, that it is most severe between the fifth and tenth years, and from the twelfth to the fifteenth year is extremely rare in any form, either trivial or severe.

The symptoms that attend the disease are by no means definite; for the functional disturbances to which it gives rise are common to it with many other affections, while the general indications of the existence of tubercular disease are often less marked in this than in other forms of phthisis. It might indeed be supposed that the tuberculous mesenteric glands would give rise, by their enlargement, to derangements similar to those which result from the diseased bronchial glands. This, however, is not the case, for the abdominal parietes, unlike those of the thorax, are soft and yielding, and allow the glands to acquire a considerable size and to approach the anterior part of the abdomen without forming adhesions with neighbouring organs. The adhesion, too, of the bronchial glands is favoured by the firm and solid character of many of the organs contained in the thorax with which they are in contact; but no such resistance is offered by the intestines, which, from the ease with which they change their position, constantly avoid compression. Hence, until the glands have acquired so large a size as to be perceptible through the parietes of the abdomen, the diagnosis of the disease is attended with much difficulty.

In the early stages of the disease there is no change in the form of the abdomen, and at a later period its increase in size is by no means an invariable occurrence; sometimes, indeed, it is retracted, and shrinks under pressure. It is true that usually as the malady advances there is some increase in the size of the abdomen; but this symptom is common to it with many other affections, and exists, as M. Guersent has remarked, as a natural condition in weak and rickety children. Even when the tuberculous glands have acquired a considerable size it is not always possible to distinguish them through the abdominal integuments. When they can be distinguished they will always be felt in the neighbourhood of the umbilicus, forming a tumour, whose surface, more or less irregular, is evidently composed of several masses agglomerated together. Their situation sometimes appears to vary, owing to the varying tension of the walls of the abdomen, according as the intestines are full or empty. The state of the tongue and bowels, the appetite, &c. and the pain which is present in the abdomen are not peculiar to *tabes*, and, consequently, render us no service in forming a diagnosis. An exception to this statement perhaps should be made with reference to the enlargement of the superficial abdominal veins, which, if there do not exist enlargement of the liver or chronic peritonitis, must at once lead to the suspicion of tubercle of the mesenteric glands.

The preceding remarks show how obscure are the local symptoms of mesenteric disease. Nor are the general symptoms less so, they being those of tuberculous disease in general, but of a rather mild character. The course of the disease is probably slow, but the obscurity of the early symptoms renders it difficult to fix the date of its commencement, and in its course it almost always becomes complicated with other forms of tuberculous disease, or else some intercurrent inflammation, as peritonitis, accelerates the fatal issue.

Nearly allied to tabes mesenterica, and not unfrequently confounded with it, is the tubercular disease of the peritoneum. Like it, too, the symptoms that betoken its existence are usually obscure. The summary of them given at p. 389, of vol. iii, we had purposed extracting, but we are prevented by want of room.

In its present form we can conscientiously recommend MM. Rilliet and Barthez's work to all who, possessing a tolerable familiarity with children's diseases are anxious to perfect their knowledge, and who will not be deterred from seeking truth by the labour of some digging to arrive at it. The book wants something, however, besides mere condensation, before it will be altogether suited for beginners. The most elaborate analysis of symptoms does not suffice to convey a correct impression of the nature of any disease. It is the talent of combining details so as to produce a vivid portraiture, that is most needed in a work intended to teach the beginner. In this, the writers have not been so successful, but we believe them to possess every qualification for the task, and we hope before long to congratulate them on having achieved it.

